# Carbon Monoxide in Pancreatic Islet Transplantation: A New Therapeutic Alternative to Patients With Severe Type 1 Diabetes Mellitus

**DOI:** 10.3389/fphar.2021.750816

**Published:** 2021-10-11

**Authors:** George J. Dugbartey

**Affiliations:** ^1^ Department of Surgery, Division of Urology, London Health Sciences Center, Western University, London, ON, Canada; ^2^ Matthew Mailing Center for Translational Transplant Studies, London Health Sciences Center, Western University, London, ON, Canada; ^3^ Multi-Organ Transplant Program, London Health Sciences Center, Western University, London, ON, Canada; ^4^ Department of Pharmacology and Toxicology, School of Pharmacy, College of Health Sciences, University of Ghana, Accra, Ghana

**Keywords:** carbon monoxide (CO), carbon monoxide-releasing molecule, pancreatic islet transplantation, type 1 diabetes mellitus, insulin therapy

## Abstract

Pancreatic islet transplantation is a minimally invasive procedure to replace β-cells in a subset of patients with autoimmune type 1 diabetic mellitus, who are extremely sensitive to insulin and lack counter-regulatory measures, and thereby increasing their risk of neuroglycopenia and hypoglycemia unawareness. Thus, pancreatic islet transplantation restores normoglycemia and insulin independence, and prevents long-term surgical complications associated with whole-organ pancreas transplantation. Nonetheless, relative inefficiency of islet isolation and storage process as well as progressive loss of islet function after transplantation due to unvoidable islet inflammation and apoptosis, hinder a successful islet transplantation. Carbon monoxide (CO), a gas which was once feared for its toxicity and death at high concentrations, has recently emerged as a medical gas that seems to overcome the challenges in islet transplantation. This minireview discusses recent findings about CO in preclinical pancreatic islet transplantation and the underlying molecular mechanisms that ensure islet protection during isolation, islet culture, transplantation and post-transplant periods in type 1 diabetic transplant recipients. In addition, the review also discusses clinical translation of these promising experimental findings that serve to lay the foundation for CO in islet transplantation to replace the role of insulin therapy, and thus acting as a cure for type 1 diabetes mellitus and preventing long-term diabetic complications.

## Introduction

Pancreatic islet transplantation is a minimally invasive procedure that serves as an alternative strategy to injected insulin therapy with the aim of restoring normoglycemia and insulin independence in a subset of patients with autoimmune type 1 diabetic mellitus without surgical complications associated with whole-organ pancreas transplantation (Shapiro et al., 2017; Rickels and Robertshon, 2019). This subpopulation of type 1 diabetic patients refers to those who are extremely sensitive to insulin and lack counter-regulatory measures, increasing their risk of neuroglycopenia (abnormally low glucose level in brain) with hypoglycemia unawareness (diabetic complication in which patient is unaware of hypoglycemia and its characteristic symptoms), severe hypoglycemic episodes and glycemic lability (swing in blood glucose from high to low and vice versa) (Shapiro et al., 2017; Rickels and Robertshon, 2019). Pancreatic islet transplantation has made great strides over the past few decades to the present era, which includes significant improvement in metabolic and safety outcomes for type 1 diabetic patients.However, a number of critical issues such as the relative inefficiency of islet isolation, islet culture, poor islet yields and progressive loss of islet function after transplantation due to unvoidable islet inflammation and apoptosis, continue to hinder a successful islet transplantation (Naftanel and Harlan, 2004; Johnson and Jones, 2012; Shapiro et al., 2017). Indeed, inflammation and apoptosis are well documented injurious events that result in the need for multiple cadaveric donor islets to achieve insulin independence (Barshes et al., 2005; Halvorsen et al., 2016; Abadpour et al., 2018).

Carbon monoxide (CO), a gas which was once feared for its toxicity and death at high concentrations, has recently emerged as a medical gas that is clinical showing promise in pancreatic islet transplantation. CO is produced in the body by heme oxygenase (HO) enzyme during heme degradation (Koçer et al., 2018; Adach and Olas, 2019). The HO enzyme exists in three isoforms: HO-1, HO-2 and HO-3 (Wu and Wang, 2005; Zobi, 2013; Kaiser et al., 2020). While HO-1 is an inducible isoform, which is expressed in response to pathological stimuli (Li et al., 2007; Leffler et al., 2011; Zhou et al., 2020), HO-2 and HO-3 are constitutive isoforms (Hayashi et al., 2004; Motterlini et al., 2005; Zobi, 2013). Low physiological concentrations of endogenous CO activates soluble guanylate cyclase (sGC), a major enzyme of the nitric oxide signaling pathway that produces cyclic guanosine monophosphate (cGMP) from guanosine triphosphate (GTP), and mediates CO-induced vasorelaxation (Ryter and Otterbein, 2004; Gomperts et al., 2017). Also, cGMP production was observed in vascular smooth muscle cells following CO treatment, and induced vasodilation and prevented hypoxia-induced cell death and sickle cell disease vaso-occlusive crises (Ryter and Otterbein, 2004; Gomperts et al., 2017).

In addition to its production in the body, CO is also administered exogenously in its gaseous form as the authentic source of exogenous CO. Besides the gaseous form, CO is also delivered exogenously into cells and tissues *via* compounds that are collectively referred to as CO-releasing molecules (CO-RMs) to ensure controlled delivery of CO into biological systems and avoid potential dangers that may arise from gaseous CO inhalation (Xue and Habtezion, 2014; Jung et al., 2020). The chemical structure of CO-RMs involves chemicals that form covalent bonds with transition metal complexes such as iron, manganese, cobalt, ruthenium, rhenium, molybdenum, giving rise to different types of CORMs such as CORM-1, CORM-2, CORM-3, CORM-307, CORM-308, CORM-314, CORM-319, CORM-401 and ALF-186 (Motterlini et al., 2005a). There is also non-metallic CO-RMs such as CORM-A1 (Motterlini et al., 2005b). Burgeoning evidence from preclinical studies shows that administration of CO-RMs during pancreatic islet isolation increases islet yields and insulin independence in type 1 diabetic transplant recipients (Nikolic et al., 2014; Nikolic et al., 2015; Cai et al., 2020). This minireview discusses current knowledge of CO gas and CO-RMs in preclinical pancreatic islet transplantation and underlying molecular mechanisms that ensure islet protection during isolation, islet culture, transplantation and post-transplant periods.

## Carbon Monoxide in Pancreatic Islet Transplantation

### Carbon Monoxide Gas in Pancreatic Islet Transplantation

**FIGURE 1:**
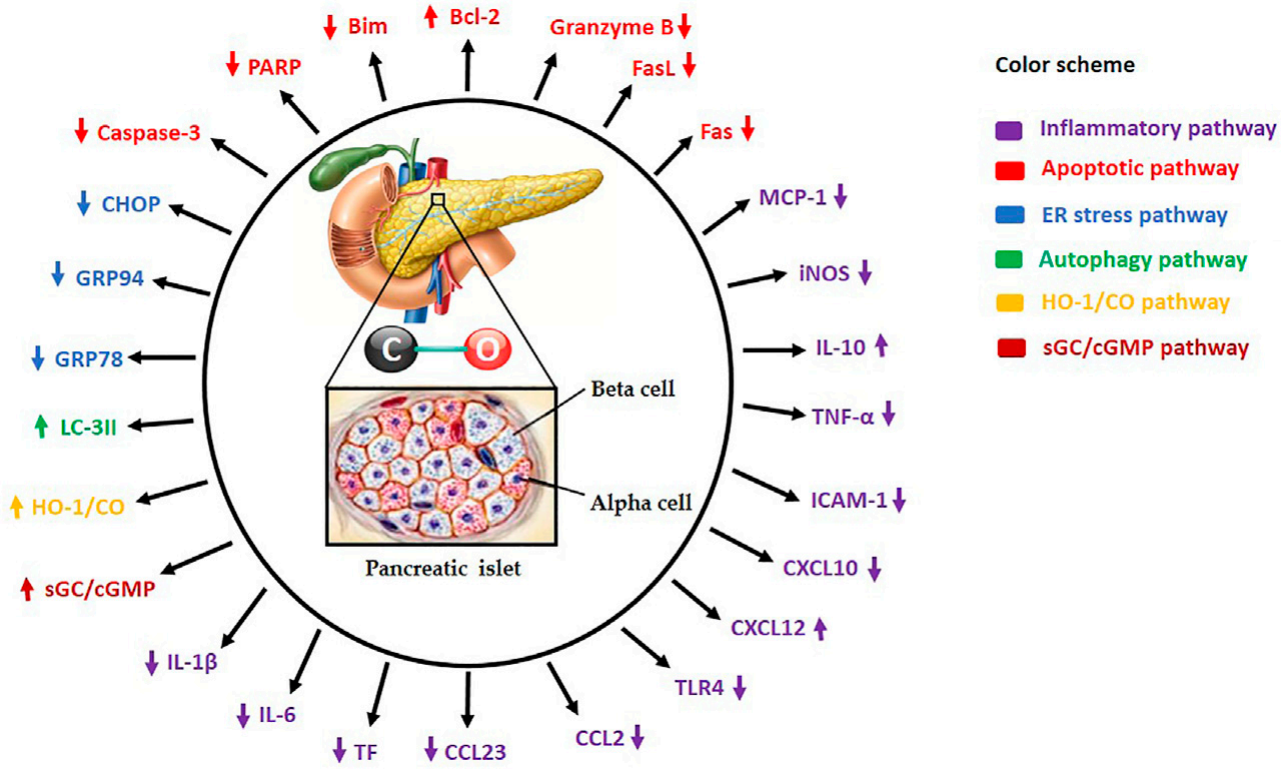
Carbon monoxide (CO) administration to islet donor and/or recipient or supplementation of standard preservation medium with CO protects and increases islet yields by modulating several molecular mechanisms. HO-1/CO: Heme oxygenase-1/carbon monoxide, sGC/cGMP: Soluble guanylate cyclase/cyclic guanosine monophosphate, TNF-α: Tumor necrosis factor-alpha, iNOS: Inducible nitric oxide synthase, IL-1β: Interleukin-1beta, IL-6: Interleukin-6, IL-10: Interleukin-10, ICAM-1: Intercellular adhesion molecule-1, Bcl-2: B-cell lymphoma-2, Caspase-3: Cysteine-aspartic proteases-3, TLR4: Toll-like receptor-4, ATP: Adenosine triphosphate, PARP: Poly-ADP ribose polymerase, MCP-1: Monocyte chemoattractant protein 1, FasL: Fas ligand, Bim: Bcl-2 interacting mediator of cell death, GRP78: Glucose-regulated protein 78, GRP94: Glucose-regulated protein 94, CHOP: C/EBP homologous protein, LC-3II: Microtubule-associated proteins 1A/1B-light chain 3B, CCL2: Chemokine (C-C motif) ligand 2, CCL23: Chemokine (C-C motif) ligand 23, CXCL10: C-X-C motif chemokine 10, CXCL12: C-X-C motif chemokine 12, and TF: Tissue factor.

In a mouse model of pancreatic allogeneic islet transplantation, exposure of healthy non-diabetic donor mice to 250 ppm CO gas for 20 h prior to islet isolation and/or 24 h before and continuously to day 13 after transplantation of 350–400 islet equivalents in each of streptozotocin (STZ; 225 mg/kg)-induced type 1 diabetic recipient mice led to markedly decreased pancreatic islet mRNA expression of pro-inflammatory genes such as tumor necrosis factor-alpha (TNF-α), inducible nitric oxide synthase (iNOS), monocyte chemoattractant protein-1 (MCP-1) and macrophage infiltration into islet grafts as well as decreased apoptotic genes such as granzyme B, Fas and Fas ligand (FasL) while simultaneously increased anti-apoptotic gene Bcl-2 significantly at various days after transplantation compared to untreated controls (Wang et al., 2005) (Figure 1). All these changes culminated in prolonged islet allograft survival following islet transplantation under the left renal capsule of diabetic recipient mice (Wang et al., 2005), which is an indication of long-term function of transplanted islet allografts and tolerance. In this experiment, the islet grafts were cultured for 24 h in CMRL medium containing 10% serum in 5% carbon dioxide at 37°C following isolation and prior to transplantation. Similarly, treatment of only the isolated islets with 1% CO-saturated medium for 24 h after isolation and before transplantation produced the same beneficial effects (Wang et al., 2005), which implies that CO administration to the donor, islet graft or recipient improves allogeneic islet survival through inhibition of inflammation and apoptotic machinery. This may also suggest that CO is able to significantly decrease the debris of dying islets that would have ordinarily triggered inflammatory response in the transplanted islets. In addition, as pro-inflammatory cytokines such as TNF-α and iNOS have direct toxic effects on pancreatic β-cells (Gunther et al., 2002), the anti-inflammatory effect of CO suggests that it is able to neutralize these toxic agents, and thus protected against β-cell death and islet rejection. Although the authors did not report on the duration of the anti-inflammatory and anti-apoptotic effects of CO after treatment, the observation that short-term CO exposure suppresses the activation of harmful signaling pathways that usually develop before and soon after transplantation and lasts for a long time is a big step forward in identifying such a mechanism. In a separate experiment by the same authors, pharmacological induction of endogenous HO-1 expression with intraperitoneal administration of 20 mg/kg of cobalt protoporphyrin-IX in healthy non-diabetic donor mice 24 h before islet harvest and/or in recipient mice every 48 h from 1 day before and after 7 days of 400–500 islet transplantation per recipient resulted in a significant upregulation of mRNA expression for HO-1 compared to islets from CO-exposed donors, and islet allografts functioned long-term compared to controls (Wang et al., 2005). This result suggests that induction of HO-1 in donor islets is an important contributing factor to the survival of islet grafts after transplantation. Previous *in vitro* and *in vivo* studies have shown that HO-1 induction in or CO treatment of monocytes and macrophages, which are commonly associated with islet destruction, enhanced anti-inflammatory effects through upregulation of anti-inflammatory IL-10 gene expression and suppressed lipopolysaccharide-induced inflammation (Inoue et al., 2001; Lee et al., 2002), which supports the observation that induction of HO-1 or activation of the HO-1/CO system in the islet or islet graft can enhance graft quality and survival after transplantation (Tullius et al., 2002; Araujo et al., 2003). It is important to note that cobalt protoporphyrin-IX is a potent inducer of HO-1 enzyme, which increases endogenous production of CO and has been used as CO substitute for protection of apoptosis in animal models of heart and liver in transplantation (Akamatsu et al., 2004; Fang et al., 2015). Interestingly, pharmacological inhibition of HO-1 activity with zinc protoporphyrin reduced endogenous CO production and reversed the cardioprotective effect of HO-1 (Akamatsu et al., 2004).

More recently, another study also examined the effect of CO gas on human and mouse islet grafts in which human islets (from donors with no history of diabetes or metabolic disorders) and mouse islets (from healthy non-diabetic donors) were placed in 1% CO-medium and cultured at 37°C in an incubator containing 250 ppm of CO gas and 1% O_2_ (hypoxic condition). Following islet culturing, mouse islet equivalent number of 150 were transplanted under the renal capsule in each of STZ (130 mg/kg)-induced type 1 diabetic recipient mice and 300 human islets transplanted into each NOD-SCID mouse (nonobese diabetic mouse with absence of T and B lymphocytes) (Kim et al., 2018). According to the authors, treatment with CO preserved the function of pancreatic β-cells at the level of insulin granule exocytosis and inhibited hypoxia-induced apoptosis through significant reduction in the expression of pro-apoptotic proteins such as caspases-3, PARP and Bim as well as pro-inflammatory proteins such as TNF-α and endoplasmic reticulum (ER) stress-related proteins (e.g. GRP78, GPR94 and CHOP), which correlated with increased expression of anti-apoptotic protein Bcl-2 (Figure 1), leading to long-term islet graft survival in type 1 diabetic recipients compared to control grafts cultured in normal medium without CO supplementation (Kim et al., 2018). Mechanistically, protection of islet integrity and function by CO was found to be partly via induction of autophagy, as the expression of autophagy marker LC-3II was upregulated in CO-treated human and mouse islets while genetic inhibition of ATG7 or ATG16L1 (two important autophagy genes) with siRNA attenuated the beneficial effects of CO (Kim et al., 2018). At the molecular level, hypoxia-induced ER stress has been reported to cause substantial loss of islet function in recipients after transplantation through interaction with mitochondrial apoptotic machinery (Bottino et al., 1998; Wang et al., 2005; Carlsson, 2011; Zheng et al., 2012). Therefore, the observation that CO protects islets from ER stress suggests that its anti-apoptotic effect is partly *via* suppression of ER stress, which also contributes to attenuating islet-directed autoimmunity and immune rejection (Wang et al., 2005). This finding supports the observation that short-term CO exposure inhibits the activation of dangerous molecular pathways that normally develop before and soon after human islet transplantation, and that CO provides significant resistance to islet cell death while maintaining insulin secretory capacity under hypoxic condition by activating an essential process that removes toxic proteins from the islet cells to maintain cellular homeostasis and function. CO has also been reported to regulate glucose-stimulated insulin release through sGC/cGMP pathway and triggers calcium transient in cells, which coordinates the secretory activity of pancreatic β-cells (Gunther et al., 2002; Lundquist et al., 2003), and further strengthens the evidence that CO increases insulin secretion and thereby improving β-cell function. Also, the observation that autophagy induced in response to hypoxia resulted in apoptosis of pancreatic β-cells while CO-induced autophagy provided islet protection is confusing. To explain this seemingly contradictory observations, although hypoxia-induced autophagy is a well-known prosurvival mechanism, it is possible that this natural mechanism is not sufficient enough to protect the islets from apoptosis, while CO-induced autophagy provides human and mouse islets with sufficient protection from hypoxia-induced apoptosis. Further studies comparing hypoxia-induced autophagy and CO-induced autophagy may be able to explain this observation.

Beyond the boundaries of animal experimentation, Wang and colleagues (2019) recently provided the first human evidence of the beneficial effects of CO gas in islet transplantation. In their randomized, controlled, double-blind pilot study, pancreatic islets were harvested from 10 non-diabetic subjects and preserved in 1% CO-saturated medium for 4 h during the isolation process and autotransplanted (138,471 ± 100,036 on the average) through the portal vein into the liver of chronic pancreatitis diabetic patients who underwent total pancreatectomy. The authors reported significantly higher islet viability before transplantation with less pancreatic β-cell death, decreased serum levels of pro-inflammatory chemokine CCL23, and increased anti-inflammatory chemokine CXCL12 at post-operative day 1 or 3 in CO-treated group compared to control islets (218,545 ± 120,422 on the average) in normal medium without CO supplementation (Wang et al., 2019) (Figure 1). In addition, 30% of the patients who received CO-treated islet grafts and none of the control subjects (0 out of 5) became insulin-independent after islet transplantation. Importantly, no adverse events directly related to CO-treated islets were observed (Wang et al., 2019), which altogether demonstrates that isolation of human islets in CO-supplemented medium improves the quality of islet grafts, and is simple and safe for chronic pancreatitis patients undergoing total pancreatectomy and islet autotransplantation, and thus increasing insulin independence in these patients. Although the authors observed reduced serum levels of CCL23 and increased CXCL12, they did not measure circulating levels of MCP-1, interleukin-8 (IL-8), TNF-α and interferon gamma (INF-γ), which are pro-inflammatory cytokines commonly observed in the blood of patients after islet transplantation. Information about the levels of these pro-inflammatory cytokines would have provided a broader picture of the protective effect of CO in human pancreatic islet transplantation. Also, CO is known to be stable in solution (e.g. the preservation medium) for only a few hours (Kohmoto et al., 2008) and hence requires the use of fresh medium. Therefore, the 4 h simple bubbling approach used by the authors to surmount this hurdle is not the most ideal procedure to ensure CO stability and its total effect on the harvested islets in the medium. A better alternative would have been the use of CO-RMs to achieve a longer CO effect. However, CO-RMs are currently not in clinical use. As this is the only clinical study done so far on this subject, more clinical studies are required to confirm and further explain the observation of the authors. Nonetheless, this promising clinical outcome suggests an effective treatment protocol with potential application not only to clinical islet autotransplantation but also to allogeneic islet transplantation. Overall, gaseous CO treatment may provide an effective pharmacological means to enhance pancreatic islet graft survival in clinical islet transplantation in type 1 diabetic patients.

### Carbon Monoxide-releasing Molecules in Pancreatic Islet Transplantation

In furtherance of the use of CO gas in pancreatic islet transplantation, a very recent study shows that CO-RMs can produce the same salutary effects as CO gas. Pretreatment with 100 μM CORM-2 during islet isolation and subsequently transplanted (150 in number) into renal subcapsular space of STZ (165 mg/kg)-induced type 1 diabetic recipient mice resulted in significant downregulation of the expression of important pro-inflammatory genes such as TNF-α, interleukin-1beta (IL-1β), IL-6, intercellular adhesion molecule-1 (ICAM-1), Toll-like receptor 4 (TLR4), tissue factor (TF), chemokine ligand 2 (CCL2) and C-X-C motif ligand 10 (CXCL10) (Figure 1), which strongly correlated with markedly reduced apoptosis, improved islet cell viability, and higher glucose-stimulated insulin secretion and transplantation outcomes in comparison with control group (Cai et al., 2020). These findings suggest that treatment of islet grafts with CORM-2 at the beginning of isolation has the potential to protect the grafts in recipients from innate inflammation and apoptosis (two important factors that account for the progressive loss of islet function after transplantation in clinical practice) from the beginning of islet harvest through peri-transplant to post-transplant periods and could improve the quality and quantity of isolated human islets. Whereas the authors did not investigate other possible effects of the anti-inflammatory and anti-apoptotic properties of CORM-2 in islet transplantation, previous reports show that pretreatment with other CO-RMs such as CORM-A1 (2 mg/kg/day) attenuates pancreatic islet-directed autoimmunity, confers protection from diabetes in mice administered with multiple low-dose STZ (40 mg/kg/day for 5 consecutive days), reduces the incidence of diabetes in nonobese diabetic mice by shifting immune response from Th1/Th17/M1 balance towards a Th2/M2 response, and induces pancreatic β-cell regeneration, and thus inhibiting inflammatory and apoptotic pathways in a mouse model of type 1 diabetes mellitus (Nikolic et al., 2014; Nikolic et al., 2015). Altogether, the anti-inflammatory and anti-apoptotic effects of CO gas and its release from CORM-2 and CORM-A1 could be exploited for clinical treatment and prevention of autoimmune type 1 diabetes mellitus and thereby preventing the need for whole-organ pancreas transplantation and insulin therapy in the future.

### Carbon Monoxide in Solid Organ Transplantation

Administration of CO gas or CO-RMs to donor, donor organ and recipient is not unique to pancreatic islet transplantation. There are reports showing cyto- and organ protection of solid organ grafts (kidney, heart, lung, liver and intestine) following administration of CO gas or CO-RMs to organ donor prior to organ procurement, donor organ during cold storage and to the organ recipient before and after transplantation (Dugbartey et al., 2021; Dugbartey, 2021a; Dugbartey, 2021b). Administration of 250 ppm of CO gas to kidney donor rats for 1 h prior to 24 h of cold storage and to recipient rats before and after kidney transplantation downregulated IL-6, IL-1β, TNF-α, ICAM-1 and iNOS and upregulated hypoxia-inducible factor-1alpha (HIF-1α) and vascular endothelial growth factor (VEGF) genes, a protective pathway that increases oxygen delivery during hypoxia. These changes resulted in significant renal graft protection and prolonged recipient survival compared air-treated control (Neto et al., 2004; Faleo et al., 2008). Vigorous bubbling of cold preservation solution with 5% compressed CO gas for 5 min before renal graft preservation also markedly inhibited the activation of pro-apoptotic and pro-inflammatory pathways, culminating in improved graft quality and recipient survival compared to control group without CO supplementation (Ozaki et al., 2012). Similarly, administration of CO-RMs such as CORM-A1, CORM-2, CORM-3 and CORM-401 also produced the same salutary effects via reduced expression of Toll-like receptors 2, 4 and 6 and activation of HO-1/CO and sGC/cGMP pathways (Sandouka et al., 2006; Bagul et al., 2008; Caumartin et al., 2011; Sener et al., 2013; Bhattacharjee et al., 2018). In the same vein, administration of CO gas and various CO-RMs resulted in graft protection and prolonged recipient survival in experimental transplantation of heart, lung, liver, and intestine through additional molecular mechanisms such as activation of NOS/cGMP, opening of mitochondrial ATP-dependent potassium (K_ATP_) channels, modulation of p38 mitogen-activated protein kinase (MAPK) signaling pathway and inhibition of the fibrotic pathway TGF-β1/Smad2 (Nakao et al., 2003; Kohmoto et al., 2006; Nakao et al., 2006; Musameh et al., 2007; Ikeda et al., 2009; Pizarro et al., 2009; Ohtsuka et al., 2014; Fujisaki et al., 2016). These promising findings provide additional mechanisms of CO-mediated graft protection with additional evidence of its clinical utility in the future.

### Clinical Translation and Challenges

Emerging experimental evidence have demonstrated that modification of current pancreatic islet transplantation protocol to include low concentrations of CO gas or CO-RMs at various steps of the protocol overcomes the challenges associated with clinical islet transplantation, leading to protection during islet isolation, islet culture, transplantation and improvement in post-transplant outcomes through modulation of several molecular mechanisms. While these results are exciting at the preclinical stage, translating them to the clinical stage is challenging, as duration of gaseous CO exposure and achieving an optimal concentration to avoid the potential danger of CO intoxication in organ donors, recipients and the transplant team are a major hurdle. Moreover, a recent study showed that replacement of CO gas with CO-RMs also poses another challenge and hinders clinical translation due to potential cytotoxic effect of CO-RMs after their degradation in the body (Motterlini et al., 2013). Hence, CO gas and CO-RMs could not be pushed forward in clinical development of improved islet transplants or other transplantable organs. In view of these challenges, organic CO-prodrugs are currently being developed to improve CO delivery into cells and tissues (Ji and Wang, 2018). Successful development of such prodrugs could push forward the clinical use of CO in transplantation of pancreatic islets and possibly solid organs and ultimately improve long-term post-transplant outcomes. In the face of these challenges, the finding that CO treatment improved islet quality and increased insulin independence following autotransplantion in chronic pancreatitis patients without any CO-related adverse events (Wang et al., 2019) is very promising. In conclusion, the therapeutic properties of CO could be exploited for clinical treatment and prevention of autoimmune type 1 diabetes mellitus and thereby preventing the need for insulin therapy and whole-organ pancreas transplantation in the future.
